# Emission-reduction cooperation among technologically complementary manufacturers under a carbon cap-and-trade mechanism with price competition

**DOI:** 10.1371/journal.pone.0345856

**Published:** 2026-03-27

**Authors:** Mingke He, Yi Zhou, Yihang Qiao, Yingzhe Ding, Xiangwei Gui

**Affiliations:** School of Business, Beijing Technology and Business University, Beijing, China; Yonsei University, KOREA, REPUBLIC OF

## Abstract

In recent years, driven by emission reduction targets, an increasing number of manufacturers producing similar products have been compelled to seek emission reduction cooperation even while competing in the market, a phenomenon that has attracted growing attention in recent studies. Based on the carbon cap-and-trade mechanism, this study develops a noncooperative–cooperative biform game model to examine the optimal decisions of technologically complementary manufacturers engaging in emission reduction cooperation under price competition. The model describes alliance profits using a characteristic function and applies the Shapley value for profit allocation, while equilibrium outcomes are derived through noncooperative game analysis. The research results show that the high carbon emission reduction investment coefficient will inhibit the carbon abatement development level of manufacturers and reduce their profits. Without a carbon cap-and-trade mechanism, competitive manufacturers lack incentives for technological collaboration. An increase in carbon trading prices significantly promotes emission reductions and profit growth, whereas the effect of government-allocated initial carbon allowances remains limited. Moreover, the improvement of manufacturers’ technological conversion capability can improve the level of carbon abatement development and enhance their profitability under price competition. In general, this study provides theoretical and practical guidance for the cooperative emission reduction of competitive enterprises under the carbon emission rights trading mechanism.

## 1. Introduction

Amid the dual pressures of global climate change and the pursuit of carbon neutrality, promoting corporate emission reduction has become a key global concern [1,2]. To achieve sustainable development, governments around the world have increasingly adopted market-based approaches, such as the carbon cap-and-trade mechanism, to encourage firms to integrate carbon emissions into their strategic decisions through economic incentives and constraints [3–5]. Under this mechanism, firms are required to purchase additional allowances when their emissions exceed the quota, while surplus allowances can be traded, making carbon emissions directly tied to production costs [6]. At the same time, the scope of regulation has expanded from the corporate level to the entire life-cycle carbon footprint of products [7,8], placing manufacturers in the production stage under stricter emission reduction obligations [9,10].

Compared with other participants in the supply chain, such as retailers, manufacturers are the main sources of carbon emissions. Because they control production processes and key technologies, they play a central role in emission reduction. However, the high costs of emission-reduction investment and increasing policy pressure make it difficult for individual firms to respond on their own. As a result, collaborative innovation and joint emission-reduction efforts have become practical choices [11–13]. For example, although Toyota, Subaru, and Mazda compete in the market, they have jointly developed low-carbon engines under the goal of carbon neutrality. Through complementary technological cooperation, they work together to meet emission regulations [14]. This suggests that technologically complementary manufacturers, who possess different yet mutually reinforcing technologies, can achieve synergy and enhance emission-reduction performance through collaboration. Driven by both policy and market forces, these manufacturers remain competitors in terms of market pricing while forming cooperative relationships in emission reduction through technological complementarity. How to optimize decision-making and balance profit under such a dual relationship deserves further study.

Most existing studies analyze corporate pricing and emission reduction using noncooperative game theory, or examine upstream–downstream collaboration through contractual mechanisms [15–19]. These studies reveal how investment costs and carbon policies influence corporate behavior, yet certain limitations remain. First, most research focuses only on the competitive relationships among manufacturers while neglecting the possibility of collaborative emission reduction. In reality, collaboration among technologically complementary manufacturers can form more efficient emission-reduction alliances, but related research is still limited. Second, existing studies tend to adopt a single-game framework, focusing either on optimal decisions under competition or on profit allocation under cooperative conditions. However, few studies provide a systematic analysis of the coexistence between price competition and emission-reduction cooperation. Therefore, there is still room to explore how cooperation can remain stable within competition and what equilibrium outcomes may arise.

In response to these limitations, this study examines how competitive manufacturers make decisions in carbon emission reduction cooperation and focuses on the following research question.

(1) How can manufacturers achieve optimal pricing and fair profit distribution in an environment that involves both competition and cooperation?
(2) How will differences in manufacturers’ technological conversion capability affect the structure of emission reduction cost sharing, and profit distribution mechanisms?
(3) How does the carbon cap-and-trade mechanism shape manufacturers’ optimal decision-making?

Building on the above research questions, this study extends existing research by refining both the analytical perspective and the description of underlying mechanisms. First, instead of treating cost sharing contracts as the core object of analysis, this study uses them as an analytical tool to represent emission reduction cooperation among manufacturers. It focuses on the internal mechanisms through which such cooperation can be achieved and sustained under continuous price competition, thereby extending existing studies on cooperation among competitive manufacturers in low carbon supply chains. Second, this study incorporates differences in manufacturers technological conversion capability to reflect heterogeneity in the efficiency with which joint emission reduction research efforts are transformed into actual emission reduction outcomes. By integrating this factor into the analytical framework of competitive emission reduction cooperation, the study provides a new mechanism based perspective for understanding behavioral differences and cooperation stability among competing manufacturers.

Therefore, this study develops a noncooperative–cooperative biform game model. A noncooperative game is used to describe the competitive relationship in the pricing stage, while a cooperative game is introduced in the emission-reduction stage to analyze alliance formation and profit allocation. At the theoretical level, this study integrates competitive pricing and cooperative emissions reduction into a unified analytical framework, revealing the logic behind emissions reduction cooperation among competitive firms and expanding the application of game theory in carbon reduction and industrial collaboration. At the practical level, the findings offer insights for enterprises to establish low-carbon cooperation mechanisms, thereby promoting the synergistic enhancement of economic efficiency and environmental performance.

The main structure of this paper is as follows. Section 2 reviews the relevant literature. Section 3 describes the research content and hypotheses. Section 4 constructs and solves the game model. Section 5 presents theorem analysis. Section 6 conducts numerical simulations. Finally, Section 7 summarizes the paper.

## 2. Literature review

This review is organized around the following research domains: cap-and-trade mechanism, Low-carbon Supply Chain and noncooperative–cooperative biform game models.

### 2.1 Carbon cap-and-trade mechanism

Existing studies mainly focus on comparing different policy instruments and examining emission-reduction structures within supply chains, but there are still some limitations in their research settings and frameworks. Huang et al. analyzed the effects of carbon taxes and carbon trading in a closed-loop supply chain and emphasized the latter’s advantage in resource allocation efficiency [20]. Eslamipoor and Sepehriyar further compared carbon taxes, government-allocated initial carbon allowances, and carbon trading, exploring their roles in promoting green supply chains. However, these studies did not deeply investigate the strategic interactions among competing firms [5]. Zou et al. incorporated corporate social responsibility into their analysis of pricing mechanisms under carbon trading, but their research was limited to a single-chain vertical structure [10]. Yavari et al. examined strategic choices in dual-channel green supply chains under carbon trading and disruptions, yet their focus remained on vertical conflicts between producers and channels [21].

Although existing research provides rich insights into single-chain, dual-channel, and multi-tier supply chain structures, there is still a lack of systematic exploration of emission-reduction cooperation among competing manufacturers under the carbon cap-and-trade mechanism. Therefore, this paper develops a game model that captures both competition and cooperation, to analyze how the carbon cap-and-trade mechanism affects the emission-reduction decisions and profit allocation of technologically complementary manufacturers through price competition and cost-sharing mechanisms. This approach offers a new perspective for understanding the role of the carbon cap-and-trade mechanism within market structures.

### 2.2 Low-carbon supply chain

Existing research on low-carbon supply chains mainly focuses on emission-reduction coordination mechanisms and game-theoretic decision-making, though there is still room to broaden the analytical perspective. In terms of contractual coordination, Yu et al. used a Stackelberg game model to investigate how cost-sharing and revenue-sharing contracts affect low-carbon cooperation within supply chains [18]. Their work mainly discusses the relative advantages of different contractual forms. Zhang et al. investigated dynamic coordination and optimization in dual-channel supply chains by incorporating reference low-carbon effects and low-carbon goodwill, but their focus remains on the effectiveness of mechanism design [22].

From the game-theory perspective, Zhu et al. applied a differential game model to analyze joint emission-reduction decisions under mixed carbon policies and growing consumer environmental awareness [23]. Li et al. adopted evolutionary game theory to explore low-carbon collaboration in the context of digital transformation, with both studies emphasizing strategic behavior within game frameworks [24]. Liu et al. compared retailer decisions under various investment scenarios and governance structures, showing that centralized supply chains improve overall profitability while simultaneously reducing emissions, though the analysis remains limited to single-scenario optimization [25].

Overall, most existing studies rely on noncooperative games and contractual mechanisms to coordinate emission-reduction behavior in supply chains, focusing mainly on strategy optimization and contract incentives. However, they rarely connect emission-reduction decisions with profit allocation in a systematic way. Moreover, most research examines vertical relationships between upstream and downstream members, with little attention to competition and cooperation among manufacturers. Therefore, this study applies a noncooperative–cooperative game approach centered on price competition and joint emission reduction, considering both decision optimization and profit allocation among manufacturers. This provides a foundation for understanding complex emission-reduction practices involving multiple interacting firms.

### 2.3 Noncooperative–cooperative biform game

In the field of supply chain coopetition, the concept of the biform game model was first introduced by Brandenburger and Stuart [26]. Since then, scholars have extended the theoretical foundations of noncooperative–cooperative biform games and applied them to analyze firms’ equilibrium strategies and benefit allocation. Regarding model refinement, Liu et al. introduced a dual-format game framework incorporating Shapley allocation functions, which enhances understanding of strategic contribution fairness by reflecting participants’ strategic choices [27]. Liu et al. further proved the existence and stability of Nash equilibrium under certain conditions within this framework [28]. In a supply chain context, Zheng et al. applied the biform game model to study eco-innovation investment mechanisms in supply chains, comparing scenarios with and without the noncooperative–cooperative framework [29]. Their analysis confirmed the superior effectiveness of the biform game model. Jia et al. focused on multi-agent reverse supply chains, incorporating a coordination mechanism for fair concern design, which essentially explores optimizing cooperative relationships in recycling and remanufacturing scenarios [30]. Zhang et al. studied the balance between collaborative investment and opportunity costs in carbon-complementary supply chains; although involving carbon elements, the core lies in investment allocation rather than emission reduction effects [31].

Most existing studies on noncooperative–cooperative biform games have mainly concentrated on building and validating theoretical models, or on examining cooperation issues in traditional contexts such as supply chain recycling and remanufacturing. However, few have explored how the dynamic interaction between competition and cooperation within supply chains affects the outcomes of low-carbon collaborative emission reduction. Therefore, this study focuses on low-carbon supply chains, aiming to extend the contextual applicability of the biform game model and provide a richer understanding of its role in low-carbon collaboration.

### 2.4 Comparative analysis with existing research

Existing research has explored emission reduction cooperation decisions and the effects of carbon policies in low carbon supply chains from multiple perspectives, including cost sharing mechanisms, joint emission reduction investment, and comparisons of different carbon policies. However, prior studies differ in their treatment of competitive environments, assumptions regarding firm heterogeneity, and approaches to analyzing the stability of emission reduction cooperation. To clarify the research position of this study and to present its distinctions from related work more clearly, Table 1 offers a systematic comparison between this study and representative literature with respect to research perspectives and mechanism description.

**Table 1:** Comparison between this study and related literature.

Reference	Competition setting	Focus of cooperation	Heterogeneity assumption	Analytical focus
Yu et al. [18]	Price competition not explicitly modeled	Comparison of cost-sharing and revenue-sharing schemes	Homogeneous firms	Contract mechanism choice
Zhang & Yu [22]	Price competition not explicitly modeled	Joint emission reduction investment and coordination	Homogeneous firms	Dynamic coordination efficiency
Liu et al. [25]	Price competition not explicitly modeled	Cooperative emission reduction investment	Homogeneous firms	Policy incentive effects
Zhu et al. [23]	Price competition not explicitly modeled	Joint emission reduction decisions	Homogeneous firms	Effects of mixed carbon policies
Huang et al. [20]	Price competition not explicitly modeled	Performance comparison of carbon policies	Homogeneous firms	Policy performance evaluation
This study	Explicit price competition (Bertrand)	Emission reduction cooperation under competitive constraints	Heterogeneous technological conversion ability	Stability of emission reduction cooperation

As indicated in Table 1, existing research mainly concentrates on emission reduction cooperation mechanisms or the performance of carbon policies themselves. Most studies examine cooperation decisions under the assumption of homogeneous firms and seldom treat price competition as an explicit constraint within the analytical framework of emission reduction cooperation. In contrast, this study explicitly considers price competition and incorporates differences in technological conversion capability among manufacturers. It emphasizes the internal mechanisms through which emission reduction cooperation can be achieved and sustained in competitive settings, thereby providing a new analytical perspective for understanding the stability of cooperation among competing manufacturers.

## 3. Problem statement and hypotheses

There are two manufacturers $M_{i}(i=1,2)$ in the supply chain producing similar products, and they compete in price in the market. Despite intense market competition, manufacturers face shared emission reduction constraints under stringent low carbon standards and policy pressures. Each manufacturer possesses a unique emission reduction technology, and these technologies are complementary. Compared with independent research efforts, joint research helps integrate the technological strengths of both parties, thereby improving the efficiency of low carbon technology innovation and reducing overall emission reduction costs [32,33]. Accordingly, the two manufacturers choose to engage in low carbon technology cooperation and establish a unified carbon abatement development level through joint research, with $M_{\text{2}}$ playing a leading role in the cooperation process.

However, due to differences in factors such as organizational learning capability and the degree of compatibility of production systems, manufacturers vary in their technological conversion capability. In particular, $M_{\text{2}}$ exhibits a higher level of technological conversion capability than $M_{\text{1}}$, which leads to asymmetry in actual emission reduction outcomes across manufacturers. During cooperation, manufacturers usually adopt cost-sharing contracts to share the expenses of emission reduction, aiming to lower overall costs and reduce profit losses caused by competition [34]. Manufacturer 1 bearing a proportion ϕ(0<ϕ<1), and Manufacturer 2 bearing the remaining share 1−ϕ.

The carbon cap-and-trade mechanism has a significant impact on firms’ emission-reduction decisions and economic performance [35–37]. The government allocates an initial carbon allowance G, to each manufacturer. The initial carbon emission per unit of product is denoted as $e_{\text{0}}$. If a manufacturer emits more or less than its allocated allowance, it can purchase or sell carbon credits in the carbon trading market at a unit carbon price $p_{e}$.

**Fig 1:**
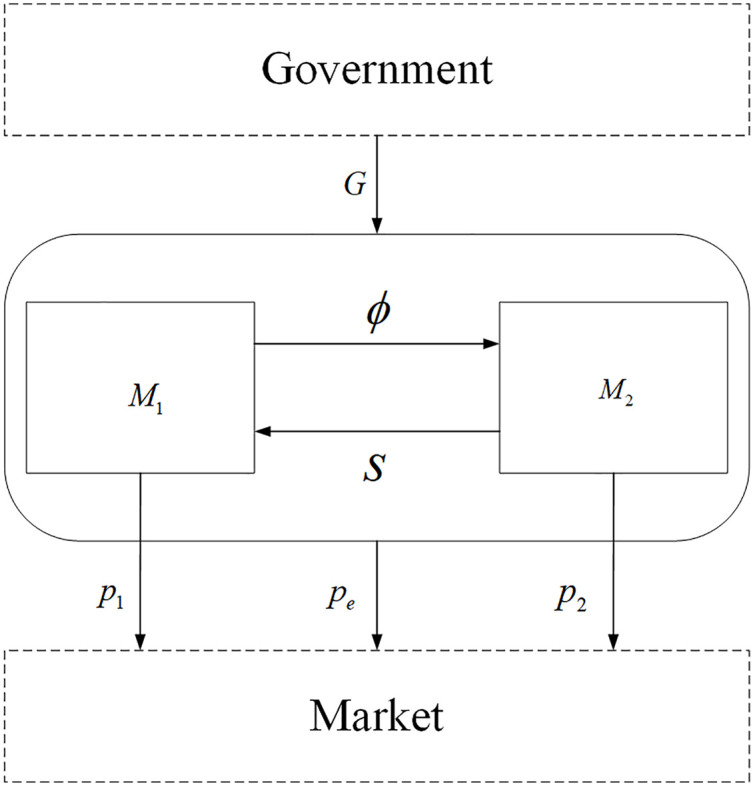
Collaborative emission reduction process in the supply chain.

To highlight the decision-making mechanisms underlying manufacturers emission reduction cooperation in competitive environments, this study does not explicitly introduce exogenously given parameters such as unit carbon footprint management costs in the model construction. Instead, the analysis focuses on endogenous factors that reflect firms decision behavior and heterogeneity. The collaborative emission reduction process within the supply chain is illustrated in Fig 1.

To facilitate the analysis, the following basic assumptions are made:

Assumption 1. Under price competition, each manufacturer i takes the product price $p_{i}$ as its decision variable. The market demand $q_{i}$ depends on both its own price $p_{i}$ and the competitor’s price $p_{j}$, i.e., $q=a-p_{i}+\alpha p_{j}$. Here, a denotes the current market share, and α∈[0,1) represents the product substitution coefficient. Both manufacturers set their prices simultaneously, forming a Bertrand competition framework [38–40].

Assumption 2. The carbon emission reduction cost invested by each manufacturer to lower product carbon emissions is $\frac{\text{1}}{\text{2}}ks^{\text{2}}$, where k is the carbon emission reduction investment coefficient. The cost of emission reduction is modeled as an increasing convex function of the reduction level, implying that as the reduction level rises, the difficulty and cost of achieving further reductions also increase [10,41].

Assumption 3. Products in the same category share high similarity in raw materials and production processes. For simplicity, the model assumes no difference in unit production costs between the two manufacturers during production [42,43].

Notations are set as shown in Table 2.

**Table 2:** Notation and definitions of key symbols.

Symbol	Description
Parameter	
$M_{i}$	Manufacturer i=1,2
a	Market capacity
c	Unit production cost
$v_{i}$	Technological conversion capability i=1,2
$q_{i}$	Market demand i=1,2
α	Product substitution coefficient
k	Carbon emission reduction investment coefficient
G	Government-allocated initial carbon allowance
$p_{e}$	Carbon trading price
$e_{\text{0}}$	Initial carbon emissions per unit product
Decision variables	
$p_{i}$	Unit product selling price i=1,2
$p_{j}$	Competitor’s unit product selling price, where j=1,2; i≠j
ϕ	Carbon reduction cost-sharing ratio
s	Carbon abatement development level

## 4. Model construction and solution of the noncooperative–cooperative biform game

**Fig 2:**
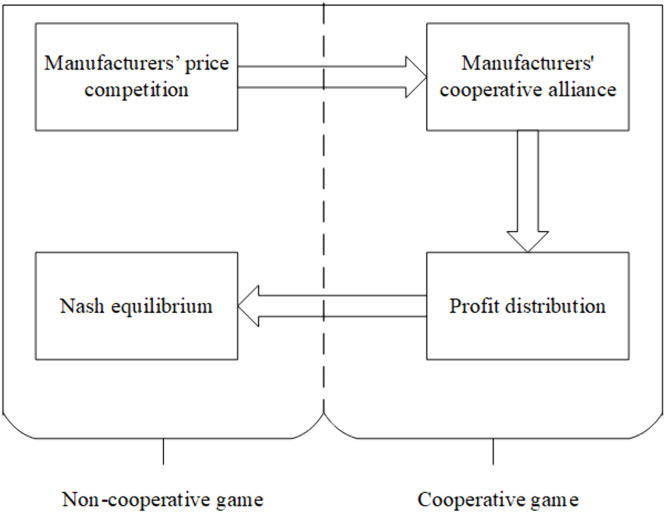
Path of the noncooperative–cooperative biform game.

This section examines the problem of price competition and collaborative emission reduction between two manufacturers, using a noncooperative–cooperative biform game approach. The detailed game structure is illustrated in Fig 2. In the noncooperative game stage, the manufacturers first engage in price competition under the Bertrand framework, where price serves as the strategic variable and any combination of competitive strategies may arise $\left(p_{\text{1}},p_{\text{2}}\right)$. Based on this stage, they then proceed to conduct a cooperative game on emission reduction. In the cooperative stage, the manufacturers jointly invest in emission reduction and share the associated costs. The players determine the carbon reduction cost-sharing ratio ϕ and the emission reduction level s. Based on different cooperative alliances, a characteristic function is constructed to calculate the profit allocation values using cooperative game theory. These profit allocation values are then used as the payoff values in the noncooperative stage, allowing for the determination of a Nash equilibrium in prices. As a result, the optimal pricing strategies $\left(\overset{*}{\underset{\text{1}}{p}},\overset{*}{\underset{\text{2}}{p}}\right)$ and the corresponding optimal profits $\overset{*}{\underset{M_{\text{1}}}{f}}\left(\overset{*}{\underset{\text{1}}{p}},\overset{*}{\underset{\text{2}}{p}}\right)$, $\overset{*}{\underset{M_{\text{2}}}{f}}\left(\overset{*}{\underset{\text{1}}{p}},\overset{*}{\underset{\text{2}}{p}}\right)$ can be derived.

According to Ji et al. and Ghosh et al. [44,45], The profit functions for manufacturers $M_{\text{1}}$ and $M_{\text{2}}$ are as follows:


$$\pi_{M_{\text{1}}}\left(p_{\text{1}},\phi ,p_{\text{2}},s\right)=\left(p_{\text{1}}-c\right)q_{\text{1}}+p_{e}G-p_{e}\left(e_{\text{0}}-v_{\text{1}}s\right)q_{\text{1}}-\phi ks^{\text{2}}/2$$ (1)

$$\pi_{M_{\text{2}}}\left(p_{\text{1}},\phi ,p_{\text{2}},s\right)=\left(p_{\text{2}}-c\right)q_{\text{2}}+p_{e}G-p_{e}\left(e_{\text{0}}-v_{\text{2}}s\right)q_{\text{2}}-(1-\phi )ks^{\text{2}}/2$$ (2)

Where $p_{e}G-p_{e}\left(e_{\text{0}}-v_{i}s\right)q_{i},(i=1,2)$ represents the manufacturer’s carbon trading cost or revenue.

### 4.1 Construction of coalition characteristic functions and profit allocation

In a non-cooperative setting, manufacturers engage in price competition and make decisions with the objective of profit maximization. Under a cooperative game framework, participants may form different coalition structures and make joint decisions within each coalition.

Although profit maximization remains the fundamental objective of manufacturers, it is necessary to further define the level of payoff that can be guaranteed under different coalition structures in a cooperative game. To this end, this study introduces a characteristic function to represent the value that each coalition can obtain when price competition still exists. In constructing the characteristic function, the analysis takes into account the possibility that external competitors may adopt unfavorable responses. Accordingly, the maximin principle is applied to determine the minimum payoff that each coalition can secure under the most adverse competitive conditions.

For any given competitive strategy profile, the characteristic value of each coalition is denoted as $v\left(p_{\text{1}},p_{\text{2}}\right)\left(A\right)$. The set of all possible coalitions is $\left\{\emptyset \right\},\left\{M_{\text{1}}\right\},\left\{M_{\text{2}}\right\},\left\{N\right\}$, where ∅ represents the empty set and N denotes the grand coalition $\left\{M_{\text{1}}M_{\text{2}}\right\}$.

The characteristic function of the empty coalition is:


$$v\left(p_{\text{1}},p_{\text{2}}\right)\left(\emptyset \right)=0$$ (3)

The characteristic function of the independent coalition $M_{\text{1}}$ is as follows:


$$\begin{array}{c} v\left(p_{\text{1}},p_{\text{2}}\right)\left(M_{\text{1}}\right)=\underset{\phi }{max}\;\underset{s}{min}\left\{\pi_{M_{\text{1}}}\left(p_{\text{1}},\phi ,p_{\text{2}},s\right)\right\}\mathrm{\backslash hfill}\\ \;=\underset{s}{min}\;\underset{\phi }{max}\left\{\begin{array}{c} @l@\left(p_{\text{1}}-c\right)\left(a-p_{\text{1}}+\alpha p_{\text{2}}\right)+p_{e}G-\\ p_{e}\left(e_{\text{0}}-v_{\text{1}}s\right)\left(a-p_{\text{1}}+\alpha p_{\text{2}}\right)-\phi ks^{\text{2}}/2 \end{array}\right\}\mathrm{\backslash hfill} \end{array}$$ (4)

As shown in the above equation, the coefficient of the carbon reduction cost-sharing ratio factor ϕ is $-\frac{\text{1}}{\text{2}}ks^{\text{2}}<0$. Therefore, when $\phi^{*}=0$, the coalition obtains the optimal solution in the sense of the max–min criterion. Substituting this into $v\left(p_{\text{1}},p_{\text{2}}\right)\left(M_{\text{1}}\right)$ yields:


$$v\left(p_{\text{1}},p_{\text{2}}\right)\left(M_{\text{1}}\right)=\underset{s}{min}\left\{\left[p_{\text{1}}-c-p_{e}\left(e_{\text{0}}-v_{\text{1}}s\right)\right]\left(a-p_{\text{1}}+\alpha p_{\text{2}}\right)+p_{e}G\right\}$$ (5)

By examining Equation (5), it can be observed that it is a first-degree function of s. For any given carbon abatement development level s, the coefficient is $p_{e}v_{\text{1}}\left(a-p_{\text{1}}+\alpha p_{\text{2}}\right)>0$, and s≥0. Therefore, the characteristic function $v\left(p_{\text{1}},p_{\text{2}}\right)\left(M_{\text{1}}\right)$ reaches its minimum when $s^{*}=0$. The characteristic function of coalition $M_{\text{1}}$ is given by:


$$v\left(p_{\text{1}},p_{\text{2}}\right)\left(M_{\text{1}}\right)=\left(p_{\text{1}}-c-p_{e}e_{\text{0}}\right)\left(a-p_{\text{1}}+\alpha p_{\text{2}}\right)+p_{e}G$$ (6)

The characteristic function of the independent coalition $M_{\text{2}}$ is as follows:


$$\begin{array}{c} v\left(p_{\text{1}},p_{\text{2}}\right)\left(M_{\text{2}}\right)=\underset{s}{max}\;\underset{\phi }{min}\left\{\pi_{M_{\text{2}}}\left(p_{\text{1}},\phi ,p_{\text{2}},s\right)\right\}\mathrm{\backslash hfill}\\ \;=\underset{s}{max}\;\underset{\phi }{min}\left\{\left(p_{\text{2}}-c\right)q_{\text{2}}+p_{e}G-p_{e}\left(e_{\text{0}}-v_{\text{2}}s\right)q_{\text{2}}-(1-\phi )ks^{\text{2}}/2\right\}\mathrm{\backslash hfill} \end{array}$$ (7)

As shown in the above equation, the coefficient of the carbon reduction cost-sharing ratio factor ϕ is $\frac{\text{1}}{\text{2}}ks^{\text{2}}>0$. Therefore, when $\phi^{*}=0$, the coalition obtains the optimal solution in the sense of the max–min criterion. Substituting this into $v\left(p_{\text{1}},p_{\text{2}}\right)\left(M_{\text{2}}\right)$ yields:


$$v\left(p_{\text{1}},p_{\text{2}}\right)\left(M_{\text{2}}\right)=\underset{s}{max}\left\{\begin{array}{c} @l@\left(p_{\text{2}}-c\right)\left(a-p_{\text{2}}+\alpha p_{\text{1}}\right)+p_{e}G-\\ p_{e}\left(e_{\text{0}}-v_{\text{2}}s\right)\left(a-p_{\text{2}}+\alpha p_{\text{1}}\right)-ks^{\text{2}}/2 \end{array}\right\}$$ (8)

By examining Equation (8), it can be observed that it is a quadratic function of s. Let $g_{\text{1}}\left(s\right)=\left(p_{\text{2}}-c\right)\left(a-p_{\text{2}}+\alpha p_{\text{1}}\right)+p_{e}G-p_{e}\left(e_{\text{0}}-v_{\text{2}}s\right)\left(a-p_{\text{2}}+\alpha p_{\text{1}}\right)-ks^{\text{2}}/2$ be given. By taking the first derivative of $g_{\text{1}}\left(s\right)$ with respect to s and setting it equal to zero, the value of $s^{*}$ under the independent alliance of $M_{\text{2}}$ can be obtained as follows:


$$s^{*}=\frac{p_{e}v_{\text{2}}\left(a-p_{\text{2}}+\alpha p_{\text{1}}\right)}{k}$$ (9)

Since $\frac{\partial^{\text{2}}g_{\text{1}}\left(s\right)}{\partial s^{\text{2}}}=-k<0$, this value maximizes $g_{\text{1}}\left(s\right)$. Substituting it into $v\left(p_{\text{1}},p_{\text{2}}\right)\left(M_{\text{2}}\right)$ yields the characteristic function of coalition $\left\{M_{\text{2}}\right\}$:


$$\begin{array}{c} v\left(p_{\text{1}},p_{\text{2}}\right)\left(M_{\text{2}}\right)=\left(p_{\text{2}}-c-p_{e}e_{\text{0}}\right)\left(a-p_{\text{2}}+\alpha p_{\text{1}}\right)+p_{e}G\mathrm{\backslash hfill}\\ +\left[\overset{\text{2}}{\underset{e}{p}}\overset{\text{2}}{\underset{\text{2}}{v}}{\left(a-p_{\text{2}}+\alpha p_{\text{1}}\right)}^{\text{2}}\right]/2k\mathrm{\backslash hfill} \end{array}$$ (10)

The characteristic function of the coalition where $M_{\text{1}}$ and $M_{\text{2}}$ cooperate is given by:


$$\begin{array}{c} v\left(p_{\text{1}},p_{\text{2}}\right)\left(N\right)=\underset{\phi ,s}{max}\left\{\pi_{M_{\text{1}}}\left(p_{\text{1}},\phi ,p_{\text{2}},s\right)+\pi_{M_{\text{2}}}\left(p_{\text{1}},\phi ,p_{\text{2}},s\right)\right\}\mathrm{\backslash hfill}\\ =\underset{\phi ,s}{max}\left\{\begin{array}{c} @l@\left(p_{\text{1}}-c\right)q_{\text{1}}+\left(p_{\text{2}}-c\right)q_{\text{2}}+2p_{e}G-\\ p_{e}\left(e_{\text{0}}-v_{\text{1}}s\right)q_{\text{1}}-p_{e}\left(e_{\text{0}}-v_{\text{2}}s\right)q_{\text{2}}-ks^{\text{2}}/2 \end{array}\right\}\mathrm{\backslash hfill} \end{array}$$ (11)

Let $g_{\text{2}}\left(s\right)=\left(p_{\text{1}}-c\right)q_{\text{1}}+\left(p_{\text{2}}-c\right)q_{\text{2}}+2p_{e}G-p_{e}\left(e_{\text{0}}-v_{\text{1}}s\right)q_{\text{1}}-p_{e}\left(e_{\text{0}}-v_{\text{2}}s\right)q_{\text{2}}-ks^{\text{2}}/2$ be given. By taking the first derivative of $g_{\text{2}}\left(s\right)$ with respect to s and setting it equal to zero, the value of $s^{*}$ the grand coalition can be obtained as follows:


$$s^{*}=\frac{p_{e}\left[v_{\text{1}}\left(a-p_{\text{1}}+\alpha p_{\text{2}}\right)+v_{\text{2}}\left(a-p_{\text{2}}+\alpha p_{\text{1}}\right)\right]}{k}$$ (12)

Since $\frac{\partial^{\text{2}}g_{\text{2}}\left(s\right)}{\partial s^{\text{2}}}=-k<0$, this value maximizes $g_{\text{2}}\left(s\right)$. Substituting it into $v\left(p_{\text{1}},p_{\text{2}}\right)\left(N\right)$ yields the characteristic function of coalition {N}:


$$\begin{array}{c} v\left(p_{\text{1}},p_{\text{2}}\right)\left(N\right)=\left(p_{\text{1}}-c-p_{e}e_{\text{0}}\right)\left(a-p_{\text{1}}+\alpha p_{\text{2}}\right)+\left(p_{\text{2}}-c-p_{e}e_{\text{0}}\right)\left(a-p_{\text{2}}+\alpha p_{\text{1}}\right)+\mathrm{\backslash hfill}\\ 2p_{e}G+\overset{\text{2}}{\underset{e}{p}}{\left[v_{\text{1}}\left(a-p_{\text{1}}+\alpha p_{\text{2}}\right)+v_{\text{2}}\left(a-p_{\text{2}}+\alpha p_{\text{1}}\right)\right]}^{\text{2}}/2k\mathrm{\backslash hfill} \end{array}$$ (13)

**Theorem 1.** The cooperative game formed by manufacturers satisfies $v\left(p_{\text{1}},p_{\text{2}}\right)\left(M_{\text{1}}\right)+v\left(p_{\text{1}},p_{\text{2}}\right)\left(M_{\text{2}}\right)\leq v\left(p_{\text{1}},p_{\text{2}}\right)\left(M_{\text{1}}⋃M_{\text{2}}\right)+v\left(p_{\text{1}},p_{\text{2}}\right)\left(M_{\text{1}}⋂M_{\text{2}}\right)$, indicating that the game exhibits both convexity and superadditivity.

The detailed proof can be found in Theorem 1 of S1 Appendix.

The core of the characteristic function is non-empty, suggesting that cooperation yields greater benefits than acting independently. In this case, no individual participant or sub-coalition has the incentive to deviate from the agreed profit allocation, ensuring the stability of the cooperative game and satisfying individual rationality. Therefore, this paper adopts the Shapley value to allocate profits based on the marginal contribution of each manufacturer. According to Zheng et al. [29], the calculation formula is as follows:


$$Sh_{i}\left(v\left(p_{\text{1}},p_{\text{2}}\right)\right)=\sum_{i\in A,A\subseteq N}\frac{\left(n-\left|A\right|\right)!\left(\left|A\right|-1\right)!}{n!}\left[v\left(p_{\text{1}},p_{\text{2}}\right)\left(A\right)-v\left(p_{\text{1}},p_{\text{2}}\right)\left(A\backslash i\right)\right]$$ (14)

Here, $i=M_{\text{1}},M_{\text{2}}$ denotes the set of all players, and n is the total number of players in the cooperative game. $\frac{\left(n-\left|A\right|\right)!\left(\left|A\right|-1\right)!}{n!}$ represents the probability of coalition A forming, while $v\left(p_{\text{1}},p_{\text{2}}\right)\left(A\right)-v\left(p_{\text{1}},p_{\text{2}}\right)\left(A\backslash i\right)$ denotes the marginal contribution of player i to coalition A. The Shapley value is interpreted as the profit allocation for player i in the cooperative game. Based on this, the profit allocations of the manufacturers after cooperation can be calculated using the following formula:


$$\begin{array}{c} f_{M_{\text{1}}}\left(p_{\text{1}},p_{\text{2}}\right)=\left(p_{\text{1}}-c-p_{e}e_{\text{0}}\right)\left(a-p_{\text{1}}+\alpha p_{\text{2}}\right)+p_{e}G+\mathrm{\backslash hfill}\\ \overset{\text{2}}{\underset{e}{p}}v_{\text{1}}\left(a-p_{\text{1}}+\alpha p_{\text{2}}\right)\left[v_{\text{1}}\left(a-p_{\text{1}}+\alpha p_{\text{2}}\right)+2v_{\text{2}}\left(a-p_{\text{2}}+\alpha p_{\text{1}}\right)\right]/4k\mathrm{\backslash hfill} \end{array}$$ (15)

$$\begin{array}{c} f_{M_{\text{2}}}\left(p_{\text{1}},p_{\text{2}}\right)=\left(p_{\text{2}}-c-p_{e}e_{\text{0}}\right)\left(a-p_{\text{2}}+\alpha p_{\text{1}}\right)+p_{e}G+\mathrm{\backslash hfill}\\ \overset{\text{2}}{\underset{e}{p}}\left\{{\left[v_{\text{1}}\left(a-p_{\text{1}}+\alpha p_{\text{2}}\right)+v_{\text{2}}\left(a-p_{\text{2}}+\alpha p_{\text{1}}\right)\right]}^{\text{2}}+\overset{\text{2}}{\underset{\text{2}}{v}}{\left(a-p_{\text{2}}+\alpha p_{\text{1}}\right)}^{\text{2}}\right\}/4k\mathrm{\backslash hfill} \end{array}$$ (16)

By comparing the Shapley values of the manufacturers with their respective standalone coalition payoffs, it can be concluded that $f_{M_{\text{1}}}\left(p_{\text{1}},p_{\text{2}}\right)-v\left(p_{\text{1}},p_{\text{2}}\right)\left(M_{\text{1}}\right)>0$ and $f_{M_{\text{2}}}\left(p_{\text{1}},p_{\text{2}}\right)-v\left(p_{\text{1}},p_{\text{2}}\right)\left(M_{\text{2}}\right)>0$. This indicates that the optimal profits of the manufacturers without cooperation are lower than the profits generated through collaboration. As a result, both manufacturers are inclined to form a coalition to reduce emission levels and pursue profit maximization.

### 4.2 Equilibrium decisions of manufacturers

The profit allocation values derived from the cooperative game serve as the payoff functions in the non-cooperative game stage. Based on this, the Nash equilibrium decisions are solved, including the optimal prices, optimal profits, emission reduction level, and Carbon reduction cost-sharing ratio.

**Theorem 2.** When $k>\frac{\overset{\text{2}}{\underset{e}{p}}}{\text{4}}[\overset{\text{2}}{\underset{\text{2}}{v}}+(\alpha v_{\text{1}}-v_{\text{2}}{)}^{\text{2}}]$, the $\overset{*}{\underset{\text{1}}{p}}$ and $\overset{*}{\underset{\text{2}}{p}}$ for manufacturers respectively are given by:


$$\overset{*}{\underset{\text{1}}{p}}=\frac{2kh_{\text{3}}\left[2kh_{\text{2}}+{p_{e}}^{\text{2}}\left(-2\overset{\text{2}}{\underset{\text{2}}{v}}-\overset{\text{2}}{\underset{\text{1}}{v}}\alpha h_{\text{1}}+v_{\text{1}}v_{\text{2}}\overset{\text{2}}{\underset{\text{1}}{h}}\right)\right]+a\left[h_{\text{7}}-2k{p_{e}}^{\text{2}}h_{\text{8}}\right]}{4k^{\text{2}}\left(4-\alpha^{\text{2}}\right)+{p_{e}}^{\text{4}}v_{\text{1}}\overset{\text{2}}{\underset{\text{2}}{v}}\left(1-\alpha^{\text{2}}\right)\left(v_{\text{1}}-2v_{\text{2}}\alpha +v_{\text{1}}\alpha^{\text{2}}\right)+4k{p_{e}}^{\text{2}}h_{10}}$$ (17)

$$\overset{*}{\underset{\text{2}}{p}}=\frac{2kh_{\text{3}}\left[2kh_{\text{2}}+{p_{e}}^{\text{2}}\left(-2\alpha \overset{\text{2}}{\underset{\text{2}}{v}}-\overset{\text{2}}{\underset{\text{1}}{v}}h_{\text{1}}+v_{\text{1}}v_{\text{2}}\overset{\text{2}}{\underset{\text{1}}{h}}\right)\right]+a\left[h_{\text{7}}-2k{p_{e}}^{\text{2}}h_{\text{9}}\right]}{4k^{\text{2}}\left(4-\alpha^{\text{2}}\right)+{p_{e}}^{\text{4}}v_{\text{1}}\overset{\text{2}}{\underset{\text{2}}{v}}\left(1-\alpha^{\text{2}}\right)\left(v_{\text{1}}-2v_{\text{2}}\alpha +v_{\text{1}}\alpha^{\text{2}}\right)+4k{p_{e}}^{\text{2}}h_{10}}$$ (18)

Substituting the optimal prices into Equation (12), s can be obtained:


$$s^{*}=\frac{2p_{e}\left(a+h_{\text{3}}h_{\text{5}}\right)\left[2k\left(v_{\text{1}}+v_{\text{2}}\right)h_{\text{2}}+\overset{\text{2}}{\underset{e}{p}}v_{\text{1}}\overset{\text{2}}{\underset{\text{2}}{v}}h_{\text{5}}h_{\text{1}}\right]}{4k^{\text{2}}\left(4-\alpha^{\text{2}}\right)+{p_{e}}^{\text{4}}v_{\text{1}}\overset{\text{2}}{\underset{\text{2}}{v}}\left(1-\alpha^{\text{2}}\right)\left(v_{\text{1}}-2v_{\text{2}}\alpha +v_{\text{1}}\alpha^{\text{2}}\right)+4k{p_{e}}^{\text{2}}h_{10}}$$ (19)

Which $h_{\text{1}}=1+\alpha$, $h_{\text{2}}=2+\alpha$, $h_{\text{3}}=c+p_{e}e_{\text{0}}$, $h_{\text{4}}=1-3\alpha -2\alpha^{\text{2}}$, $h_{\text{5}}=\alpha -1$, $h_{\text{6}}=\alpha -2$, $h_{\text{7}}=4k^{\text{2}}h_{\text{2}}+\overset{\text{4}}{\underset{e}{p}}v_{\text{1}}\overset{\text{2}}{\underset{\text{2}}{v}}h_{\text{1}}[v_{\text{1}}(1+\alpha^{\text{2}})-2\alpha v_{\text{2}}]$, $h_{\text{8}}=2\overset{\text{2}}{\underset{\text{2}}{v}}h_{\text{1}}+\overset{\text{2}}{\underset{\text{1}}{v}}h_{\text{2}}+v_{\text{1}}v_{\text{2}}h_{\text{4}}$, $h_{\text{9}}=\overset{\text{2}}{\underset{\text{1}}{v}}+2\overset{\text{2}}{\underset{\text{2}}{v}}h_{\text{2}}+v_{\text{1}}v_{\text{2}}h_{\text{4}}$, $h_{10}=\left[-{v_{\text{1}}}^{\text{2}}+v_{\text{1}}v_{\text{2}}\alpha \left(3-\alpha^{\text{2}}\right)-\overset{\text{2}}{\underset{\text{2}}{v}}\left(2-\alpha^{\text{2}}\right)\right]$.

By substituting $\overset{*}{\underset{i}{p}}(i=1,2)$ into Equations (15) and(16), the optimal profits $\overset{*}{\underset{M_{\text{1}}}{\pi }}$ and $\overset{*}{\underset{M_{\text{2}}}{\pi }}$ for the players can be derived. Then, by equating the profit functions of $\pi_{M_{\text{1}}}\left(p_{\text{1}},\phi ,p_{\text{2}},s\right)$ and $\overset{*}{\underset{M_{\text{1}}}{\pi }}$, $\phi^{*}$ can be obtained. Since the expressions for $\overset{*}{\underset{M_{\text{1}}}{\pi }}$, $\overset{*}{\underset{M_{\text{2}}}{\pi }}$, and $\phi^{*}$ are relatively complex.

The detailed proof can be found in Theorem 2 of S1 Appendix.

## 5. Analysis of the influencing factors on equilibrium decisions

During the production process, manufacturers production costs and technological conversion capability play an important role in shaping equilibrium decisions among participants. Therefore, this section focuses on analyzing how these parameters affect $\left(\overset{*}{\underset{\text{1}}{p}},\overset{*}{\underset{\text{2}}{p}}\right)$ and $s^{*}$.Since the optimal solutions for profit and the carbon reduction cost-sharing ratio are relatively complex, a detailed numerical simulation analysis will be presented in Section 6.

**Theorem 3.** When $k>\frac{\overset{\text{2}}{\underset{e}{p}}\left[\overset{\text{2}}{\underset{\text{1}}{v}}+v_{\text{1}}v_{\text{2}}\alpha \left(\alpha^{\text{2}}-3\right)-\overset{\text{2}}{\underset{\text{2}}{v}}\left(\alpha^{\text{2}}-2\right)\right]}{4-\alpha^{\text{2}}}$, $\frac{\partial \overset{*}{\underset{\text{1}}{p}}}{\partial c}>0$, $\frac{\partial \overset{*}{\underset{\text{2}}{p}}}{\partial c}>0$, $\frac{\partial s^{*}}{\partial c}<0$.

The detailed proof can be found in Theorem 3 of S1 Appendix.

According to Theorem 3, when $k>\frac{\overset{\text{2}}{\underset{e}{p}}\left[\overset{\text{2}}{\underset{\text{1}}{v}}+v_{\text{1}}v_{\text{2}}\alpha \left(\alpha^{\text{2}}-3\right)-\overset{\text{2}}{\underset{\text{2}}{v}}\left(\alpha^{\text{2}}-2\right)\right]}{4-\alpha^{\text{2}}}$, an increase in unit production cost leads to higher optimal selling prices and a lower optimal level of carbon abatement development. A high carbon emission reduction investment coefficient implies that a large amount of research input is required for each unit of emission reduction, making abatement activities highly sensitive to cost changes. Under this condition, rising production costs not only directly compress manufacturers profit margins but also significantly increase the marginal cost of continuing carbon abatement research investment. As a result, manufacturers are more inclined to transfer part of the cost pressure to the market by raising product prices rather than maintaining the original intensity of carbon abatement development. At the same time, although the carbon trading mechanism provides firms with some flexibility, the rigidity of abatement investment costs becomes dominant under high abatement investment conditions, which causes the optimal carbon abatement level to decline as production costs increase.

**Theorem 4.** When $k>k_{v_{\text{1}}}$, $\frac{\partial s^{*}}{\partial v_{\text{1}}}>0$; When $k>k_{v_{\text{2}}}$, $\frac{\partial s^{*}}{\partial v_{\text{2}}}>0$.

The detailed proof can be found in Theorem 4 of S1 Appendix in S1 File.

According to Theorem 4, when the carbon emission reduction investment coefficient exceeds a threshold, improvements in manufacturers technological conversion capability lead to an increase in the carbon abatement development level. Under a high carbon abatement investment coefficient, joint emission reduction research faces substantial initial investment pressure. However, manufacturers technological conversion capability determines the efficiency with which research investment is transformed into actual emission reduction outcomes. When the technological conversion capability of either manufacturer improves, the carbon abatement technology generated through joint research can be translated more effectively into realized emission reduction, thereby reducing the effective cost per unit of abatement. Because emission reduction technologies are complementary, this efficiency improvement benefits not only the manufacturer with enhanced capability but also improves overall abatement performance through the joint research mechanism. As a result, even when manufacturers differ in technological conversion capability, an improvement by either party promotes a higher optimal carbon abatement development level.

## 6. Numerical analysis

This section uses numerical simulation to run dynamic experiments on manufacturers’ equilibrium decisions, and it examines how parameters—such as the carbon emission reduction investment coefficient and the carbon trading price—affect optimal decisions including price and profit. Following Zhou et al., Li et al., and Wu et al. [46–48], the model parameters are set to a=1000, c=5, $v_{\text{1}}=0.6$, $v_{\text{2}}=0.8$, α=0.8, k=3, G=500. In addition, because the carbon trading price changes with market dynamics, and for ease of statistical analysis, we follow Wu et al. and Zou et al. [49,50] and set the carbon trading price to $p_{e}=1$.

### 6.1 Effects of the carbon emission reduction investment coefficient on optimal decisions

**Fig 3:**
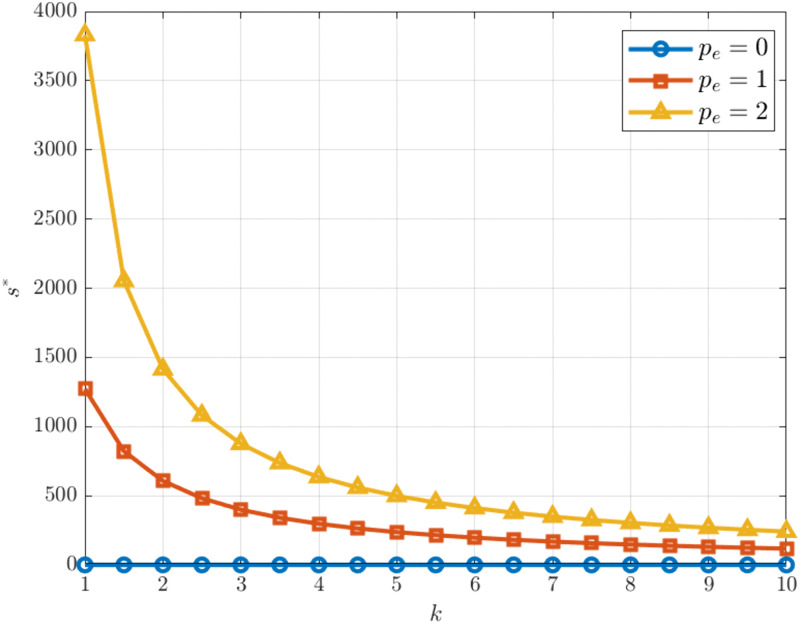
$s^{*}$ vs. k under different. $p_{e}$.

As shown in Fig 3, carbon abatement development level decreases as the carbon emission reduction investment coefficient increases under different carbon trading price levels. This result is consistent with the findings of Kang et al. [51], who reported that a higher abatement cost coefficient significantly discourages participants’ investment in emission reduction efforts. A further comparison across carbon trading prices shows that higher prices substantially raise the optimal emission reduction level, suggesting that the carbon trading mechanism effectively encourages enterprises to reduce emissions through price signals.

However, as the investment coefficient continues to rise, the gap between the curves gradually narrows. This indicates that when emission-reduction investments become overly expensive, even higher carbon trading prices provide a weaker incentive. In addition, when the carbon trading price is zero, the emission reduction level is close to its minimum, showing that without the incentive of carbon trading, firms are unlikely to invest in emission reduction activities.

**Fig 4:**
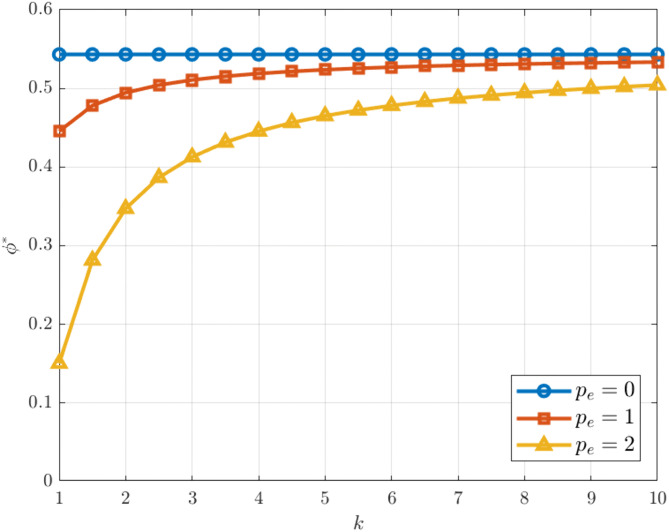
$\phi^{*}$ vs. k under different. $p_{e}$.

Fig 4 shows that under different carbon trading price levels, the optimal cost-sharing ratio for carbon emission reduction generally increases and then stabilizes as the carbon reduction investment coefficient rises. This suggests that in high-cost environments, cost sharing among enterprises gradually becomes fixed, leading to a more balanced and stable allocation pattern. A comparison across various carbon trading price scenarios indicates that when $p_{e}=0$, $\phi^{*}$ remains consistently high and is barely affected by k; when $p_{e}=1$, the sharing ratio is slightly lower but gradually converges as k increases; whereas under higher carbon trading price conditions, the initial sharing ratio is the lowest but rises rapidly with increasing k, eventually approaching the levels observed in other scenarios. These results imply that higher carbon trading prices amplify differences in cost-sharing ratios at low-cost stages but weaken their moderating effect as costs continue to rise.

**Fig 5:**
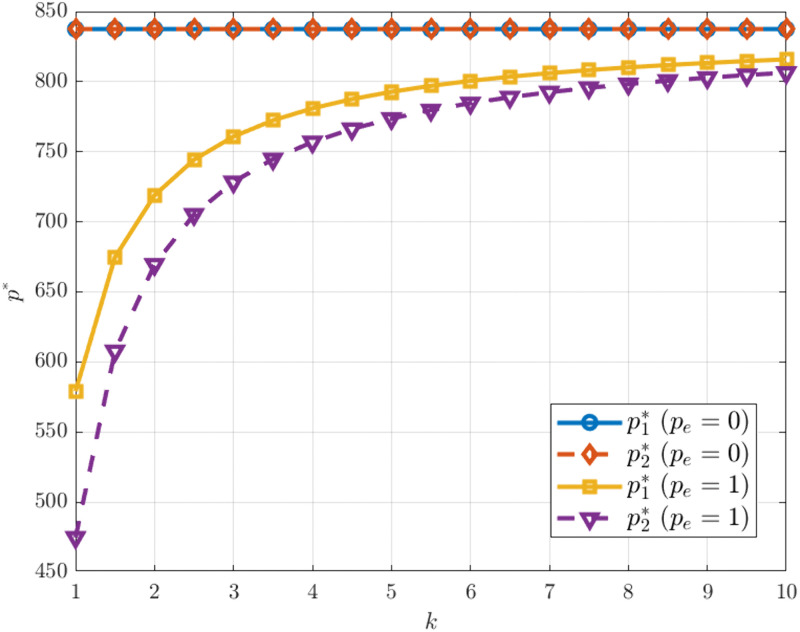
$p^{*}$ vs. k under different. $p_{e}$.

Fig 5 shows how the optimal prices of the two manufacturers change with the carbon emission reduction investment coefficient under different carbon trading price conditions. The results indicate that in the absence of carbon trading, both manufacturers maintain high and relatively stable optimal prices, which are almost unaffected by changes in k.This suggests that without carbon price constraints, the cost of emission reduction investments has only a limited impact on firms’ pricing strategies. In contrast, when a carbon trading mechanism is in place, the optimal price gradually rises with increasing k and eventually stabilizes. This finding is consistent with Wang et al. [52], who pointed out that a higher carbon emission reduction investment coefficient increases firms’ marginal production costs. Such cost pressure is typically passed on to consumers through price adjustments, ultimately resulting in higher product prices.

**Fig 6:**
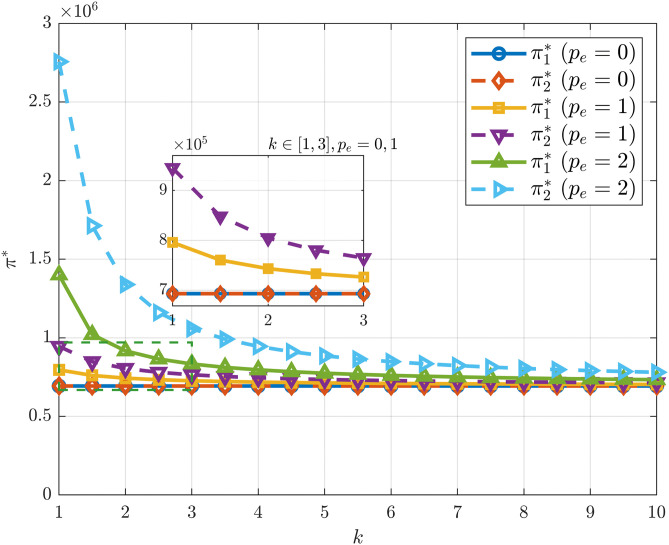
π^*^ vs. k under different. $p_{e}$.

Fig 6 further shows that under different carbon trading price levels, the profit decreases as the carbon emission reduction investment coefficient increases. Similarly, Xu et al. [53], drawing on multiple studies, found that as the abatement cost coefficient rises, the profits of supply chain members and the system as a whole tend to decline. When the carbon trading price is zero, corporate profits remain consistently low, showing almost no variation with k.This suggests that in the absence of carbon trading incentives, emission reduction provides no additional revenue for manufacturers, leaving both firms with little motivation to cooperate.

When a carbon trading mechanism is present, profits decline rapidly as k increases. Under high carbon trading price conditions, profits are initially the highest, particularly for firms with higher technological conversion capability, which gives them a noticeable advantage. However, as k continues to grow, this advantage gradually narrows and eventually converges to the levels observed under medium- and low-carbon trading price conditions in high-cost regions. Overall, these results indicate that high carbon trading prices can effectively encourage firms to enhance profits in low-cost settings. Yet under high-cost conditions, the incentive effect of carbon trading becomes insufficient to offset the increasing cost pressures.

### 6.2 Effects of the carbon cap-and-trade mechanism on optimal decisions

**Fig 7:**
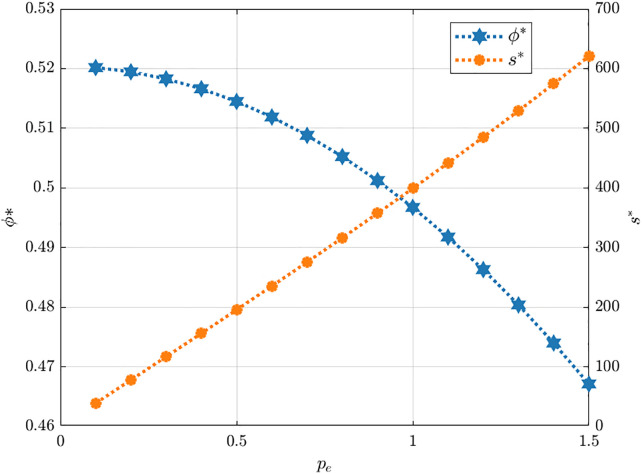
The influence of $p_{e}$ on $s^{*}$. $\phi^{*}$.

As shown in Fig 7, rising carbon prices elevate the optimal carbon abatement development level while lowering carbon reduction cost-sharing ratio. Higher carbon prices enhance the value of each emission allowance, prompting manufacturers to increase their emission reduction levels to save costs or capture potential carbon revenues, this finding consistent with the research by Wang et al. [54]. Moreover, greater abatement efforts inevitably lead to higher total costs. Ji et al. and Wang et al. [52,55] found that under stronger carbon cost pressures, excessively high cost-sharing ratios are detrimental to coordination and profit. Building on this, we find that firms with higher technological conversion capability tend to voluntarily assume a greater share of the costs in order to maintain stable cooperation and sustain their gains from the carbon market. This explains the observed downward trend in the optimal cost-sharing ratio as carbon trading prices rise.

**Fig 8:**
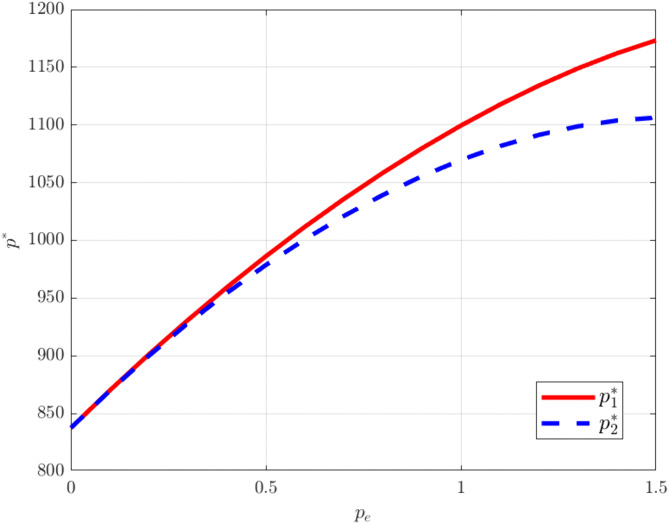
The influence of $p_{e}$ on. $\overset{*}{\underset{i}{p}}$.

**Fig 9:**
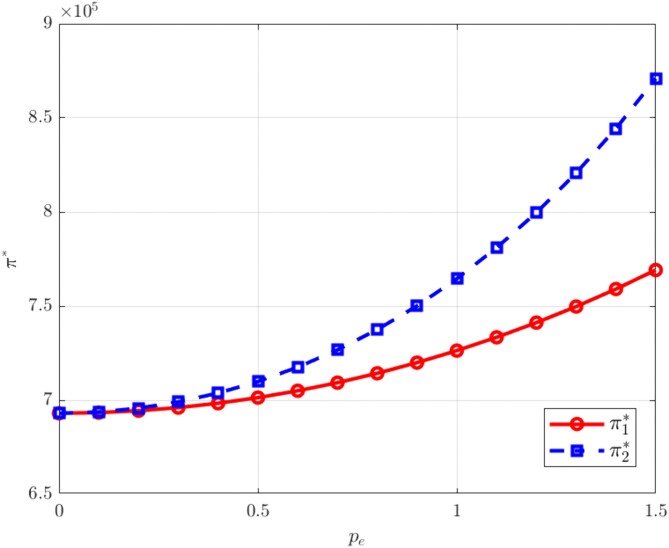
The influence of $p_{e}$ on. $\overset{*}{\underset{i}{\pi }}$.

As shown in Fig 8–9, rising carbon trading prices lead to increases in both product prices and corporate profits. Under the carbon trading mechanism, an increase in the carbon trading price raises firms’ marginal production costs. Because manufacturers must purchase additional emission allowances in the market at higher costs, these implicit expenses are passed on to product prices, driving them upward. At the same time, higher carbon trading prices also enhance the market value of carbon allowances. Through joint research and development and cost-sharing, the two manufacturers effectively buffer individual cost pressures. Under price competition, they are able to maintain market equilibrium by coordinating price increases, allowing the rate of price growth to exceed the rise in costs and thereby generating additional profits.

**Fig 10:**
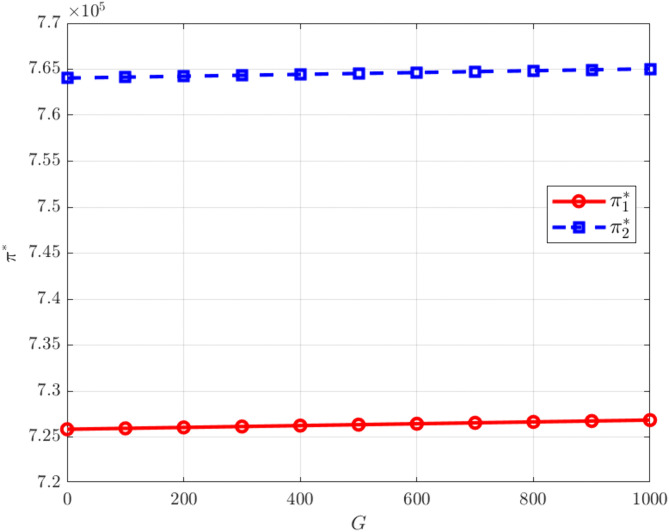
The influence of G on. $\overset{*}{\underset{i}{\pi }}$.

As shown in Fig 10, an increase in the initial carbon allowance allocated by the government leads to higher profits for manufacturers. A larger allowance means that manufacturers can use more emission credits freely during production, reducing their dependence on external carbon markets and lowering carbon trading expenses. In addition, when the actual emissions achieved through cooperative emission reduction are below the allocated quota, the surplus allowances can be sold on the carbon market, creating additional trading income. Together, these mechanisms allow higher carbon allowances to both reduce production costs and broaden profit channels, ultimately enhancing firms’ overall profitability.

### 6.3 Effects of technological conversion capability on optimal decisions

**Fig 11:**
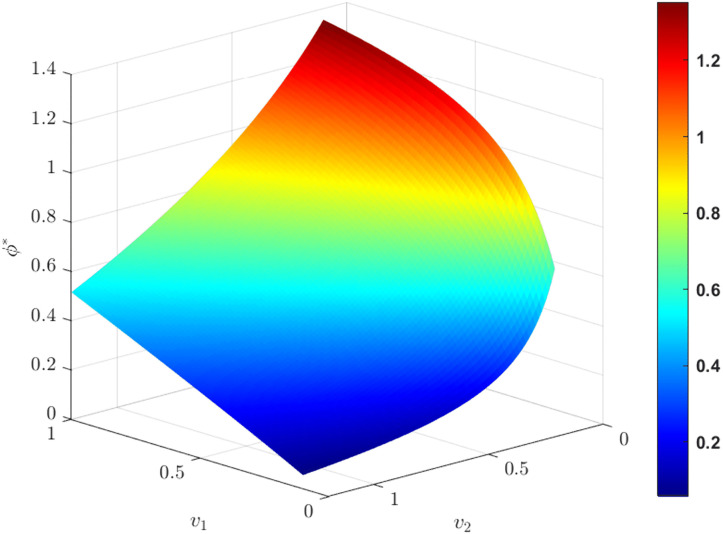
The influence of $v_{i}(i=1,2)$ on. $\phi^{*}$.

As shown in Fig 11, when the technological conversion capability of $M_{\text{1}}$ increases, Carbon reduction cost-sharing ratio rises. In contrast, an increase in the technological conversion capability of $M_{\text{2}}$ leads to an opposite trend. When the carbon abatement development level remains unchanged, an improvement in manufacturers technological conversion capability implies higher realized emission reduction performance, allowing manufacturers to save more carbon related costs or generate additional carbon related revenues. To maintain cooperation stability, manufacturers with improved technological conversion capability are therefore willing to bear a larger share of emission reduction costs.

**Fig 12:**
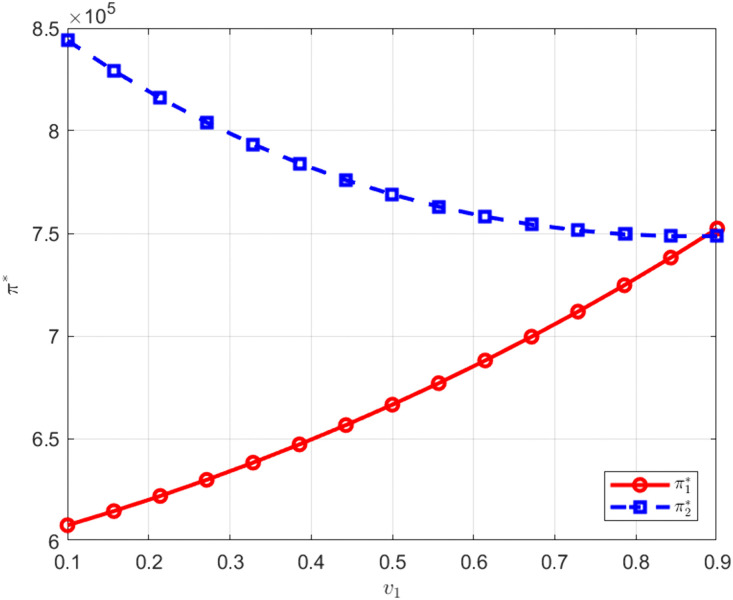
The influence of $v_{\text{1}}$ on. $\pi^{*}$.

**Fig 13:**
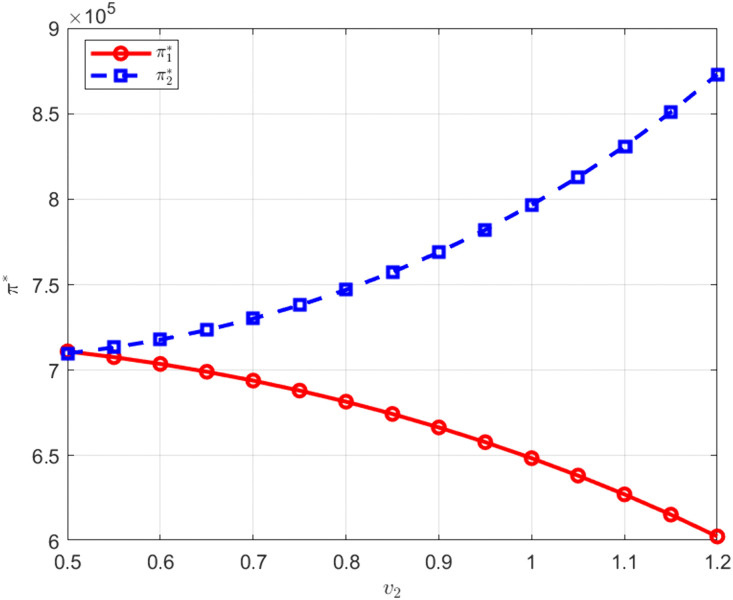
The influence of $v_{\text{2}}$ on. $\pi^{*}$.

As shown in Fig 12 and 13, when the technological conversion capability of $M_{\text{1}}$ increases, the profit of $M_{\text{1}}$ rises while the profit of $M_{\text{2}}$ declines. When the technological conversion capability of $M_{\text{2}}$ improves, the opposite pattern of profit change is observed. Because manufacturers establish a unified carbon abatement development level through joint research, technological conversion capability determines the efficiency with which cooperative outcomes are transformed into economic returns. An improvement in technological conversion capability reduces the effective cost per unit of abatement or increases carbon trading revenue, thereby strengthening a manufacturer relative cost advantage in price competition equilibrium and raising its profit. Under a given demand structure, this advantage is transmitted through price competition and compresses the profit space of the other manufacturer, reflecting a redistribution effect of competitive gains within the context of cooperative research.

## 7. Conclusion

Under the carbon cap-and-trade mechanism, this study develops a noncooperative–cooperative biform game model to analyze price competition and emission reduction cooperation between two manufacturers.

### 7.1 Research findings

First, this study finds that manufacturers technological conversion capability plays a critical role in emission reduction cooperation. An improvement in technological conversion capability enhances the efficiency with which joint research outcomes are transformed into actual emission reduction effects, thereby promoting a higher optimal carbon abatement development level under a high carbon emission reduction investment coefficient. Manufacturers with stronger technological conversion capability exhibit greater profitability in price competition equilibrium and drive a redistribution of cooperative gains between manufacturers.

Second, the carbon emission reduction investment coefficient has a significant impact on manufacturers strategic choices. As emission reduction investment intensity increases, the rigidity of abatement costs becomes stronger, and manufacturers mainly transmit cost pressure to the market by raising product prices, which leads to a decline in both the optimal carbon abatement development level and profit levels. In this context, the carbon trading price can effectively mitigate the negative effects of high abatement costs on emission reduction incentives and cooperation stability, while the Government allocated initial carbon allowance has a relatively limited influence on emission reduction incentives.

Finally, when price competition and emissions reduction cooperation coexist, manufacturers can achieve a stable equilibrium in pricing and carbon reduction decisions through joint research and cost-sharing mechanisms, verifying the effectiveness of the noncooperative–cooperative biform game theory in coordinating emissions reduction behavior in competitive supply chains.

### 7.2 Research implications

In practice, it is necessary to develop targeted low-carbon strategies based on the differences between enterprises. Manufacturers with stronger technology transfer capabilities tend to play a leading role in joint emission-reduction research and development, and are more willing to increase both their emission-reduction investment and cost-sharing share, thereby reinforcing their cost advantage in price competition and sustaining cooperative stability. Manufacturers with weaker technology transfer capabilities should actively participate in emissions reduction cooperation, leveraging technology spillovers and carbon trading revenue to improve emissions reduction efficiency and gradually enhance their own technology absorption and transformation capabilities, avoiding continuous squeeze on profit margins in competition. This study provides practical insights for competitive manufacturers seeking emissions reduction cooperation, reasonable emissions reduction cooperation and carbon trading mechanisms can jointly improve economic performance and environmental benefits.

### 7.3 Limitations and future research directions

While this study provides a new perspective on game theory in low-carbon supply chains, it still has some limitations. First, the analysis focuses primarily on manufacturers and does not adequately consider the strategic responses of upstream and downstream enterprises in the supply chain. Future research could expand the framework to include multiple actors or hierarchical structures to examine how supply chain participants compete and cooperate under dynamic carbon policies and fluctuating market demand. Furthermore, this study emphasizes the impact of carbon trading mechanisms on supply chain decision-making. Future research could incorporate comparative analyses of other policy tools to assess their respective impacts on emissions reduction and profit optimization.

## Supporting information

S1 File Appendix. (DOCX)
