# Supplementary material for: Emission-reduction cooperation among technologically complementary manufacturers under a carbon cap-and-trade mechanism with price competition
Source: PLoS One. 2026 Mar 27;21(3):e0345856. doi: 10.1371/journal.pone.0345856 (PMC13028506; doi:10.1371/journal.pone.0345856)
Supplement: S1 File — (DOCX) [file pone.0345856.s001.docx]

# S1 Appendix

## Proof of Theorem 1:

According to equations (6), (10), and (13), we have:

$$v\left(p_{1},p_{2}\right)\left(M_{1}\cup M_{2}\right)-v\left(p_{1},p_{2}\right)\left(M_{1}\right)-v\left(p_{1},p_{2}\right)\left(M_{2}\right)=\frac{p_{e}^{2}v_{1}q_{1}(v_{1}q_{1}+2v_{2}q_{2})}{2k}\geq0$$

Therefore，$v\left(p_{1},p_{2}\right)\left(M_{1}\right)+v\left(p_{1},p_{2}\right)\left(M_{2}\right)\leq v\left(p_{1},p_{2}\right)\left(M_{1}\cup M_{2}\right)+v\left(p_{1},p_{2}\right)\left(M_{1}\cap M_{2}\right)$.

## Proof of Theorem 2:

Let the payoffs of manufacturers$M_{1}$and$M_{2}$, denoted as$f_{M_{1}}\left(p_{1},p_{2}\right)$​and$f_{M_{2}}\left(p_{1},p_{2}\right)$, be differentiated twice with respect to the price. The second-order derivatives are given by:

$$\frac{\partial^{2}f_{M_{1}}(p_{1},p_{2})}{\partial p_{1}^{2}}=\frac{-8k+p_{e}^{2}[2v_{2}^{2}+2(\alpha v_{1}-v_{2})^{2}]}{4k}$$

$$\frac{\partial^{2}f_{M_{2}}(p_{1},p_{2})}{\partial p_{2}^{2}}=\frac{-4k-p_{e}^{2}v_{1}(2\alpha v_{2}-v_{1})}{2k}$$

When $k>\frac{p_{e}^{2}}{4}[v_{2}^{2}+(\alpha v_{1}-v_{2})^{2}]$, $\frac{\partial^{2}f_{M_{1}}(p_{1},p_{2})}{\partial p_{1}^{2}}<0$and$\frac{\partial^{2}f_{M_{2}}(p_{1},p_{2})}{\partial p_{2}^{2}}<0$, there exists an optimal price that maximizes the profits of the supply chain members. Next, taking the first-order partial derivatives of$f_{M_{1}}\left(p_{1},p_{2}\right)$​and$f_{M_{2}}\left(p_{1},p_{2}\right)$with respect to the price, and setting them equal to zero, we obtain:

$$\left\{\begin{matrix} \frac{\partial f_{M_{1}}(p_{1},p_{2})}{\partial p_{1}}=0 \\ \frac{\partial f_{M_{2}}(p_{1},p_{2})}{\partial p_{2}}=0 \end{matrix}\right.$$

By solving the system of equations simultaneously, the optimal price can be obtained as follows:

$$p_{1}^{*}=\frac{2kh_{3}[2kh_{2}+p_{e}^{2}(-2v_{2}^{2}-v_{1}^{2}\alpha h_{1}+v_{1}v_{2}h_{1}^{2})]+a[h_{7}-2kp_{e}^{2}h_{8}]}{4k^{2}(4-\alpha^{2})+p_{e}^{4}v_{1}v_{2}^{2}(1-\alpha^{2})(v_{1}-2v_{2}\alpha+v_{1}\alpha^{2})+4kp_{e}^{2}h_{10}}$$

$$p_{2}^{*}=\frac{2kh_{3}[2kh_{2}+p_{e}^{2}(-2\alpha v_{2}^{2}-v_{1}^{2}h_{1}+v_{1}v_{2}h_{1}^{2})]+a[h_{7}-2kp_{e}^{2}h_{9}]}{4k^{2}(4-\alpha^{2})+p_{e}^{4}v_{1}v_{2}^{2}(1-\alpha^{2})(v_{1}-2v_{2}\alpha+v_{1}\alpha^{2})+4kp_{e}^{2}h_{10}}$$

Which$h_{1}=1+\alpha$,$h_{2}=2+\alpha$,$h_{3}=c+p_{e}e_{0}$,$h_{4}=1-3\alpha-2\alpha^{2}$,$h_{5}=\alpha-1$,$h_{6}=\alpha-2$,$h_{7}=4k^{2}h_{2}+p_{e}^{4}v_{1}v_{2}^{2}h_{1}[v_{1}(1+\alpha^{2})-2\alpha v_{2}]$,$h_{8}=2v_{2}^{2}h_{1}+v_{1}^{2}h_{2}+v_{1}v_{2}h_{4}$,$h_{9}=v_{1}^{2}+2v_{2}^{2}h_{2}+v_{1}v_{2}h_{4}$,$h_{10}=[-v_{1}^{2}+v_{1}v_{2}\alpha(3-\alpha^{2})-v_{2}^{2}(2-\alpha^{2})]$.

Substituting the optimal price$\left(p_{1}^{*},p_{2}^{*}\right)$into the equation (12) yields the optimal carbon emission reduction level:

$$s^{*}=\frac{2p_{e}(a+h_{3}h_{5})[2k(v_{1}+v_{2})h_{2}+p_{e}^{2}v_{1}v_{2}^{2}h_{5}h_{1}]}{4k^{2}(4-\alpha^{2})+p_{e}^{4}v_{1}v_{2}^{2}(1-\alpha^{2})(v_{1}-2v_{2}\alpha+v_{1}\alpha^{2})+4kp_{e}^{2}h_{10}}$$

By substituting$\left(p_{1}^{*},p_{2}^{*}\right)$ into the payoff functions of$f_{M_{1}}\left(p_{1},p_{2}\right)$​and$f_{M_{2}}\left(p_{1},p_{2}\right)$, respectively, the optimal profits for the players can be obtained as follows:

By combining the profit functionsand$\pi_{M_{1}}^{*}$of manufacturer $M_{1}$​, the expression forcan be derived as follows:

## Proof of Theorem 3:

Compute the first-order partial derivatives of the optimal price with respect to $c$, respectively:

$$\frac{\partial p_{1}^{*}}{\partial c}=\frac{2k[2kh_{2}+p_{e}^{2}(-2v_{2}^{2}-v_{1}^{2}\alpha h_{1}+v_{1}v_{2}h_{1}^{2})]}{4k^{2}(4-\alpha^{2})+p_{e}^{4}v_{1}v_{2}^{2}(1-\alpha^{2})(v_{1}-2v_{2}\alpha+v_{1}\alpha^{2})+4kp_{e}^{2}h_{10}}$$

$$\frac{\partial p_{2}^{*}}{\partial c}=\frac{2k[2kh_{2}+p_{e}^{2}(-2v_{2}^{2}\alpha-v_{1}^{2}h_{1}+v_{1}v_{2}h_{1}^{2})]}{4k^{2}(4-\alpha^{2})+p_{e}^{4}v_{1}v_{2}^{2}(1-\alpha^{2})(v_{1}-2v_{2}\alpha+v_{1}\alpha^{2})+4kp_{e}^{2}h_{10}}$$

If the conditions$k>\frac{p_{e}^{2}\left[v_{1}^{2}+v_{1}v_{2}\alpha\left(\alpha^{2}-3\right)-v_{2}^{2}\left(\alpha^{2}-2\right)\right]}{4-\alpha^{2}}$​and are satisfied, then$\frac{\partial p_{1}^{*}}{\partial c}>0$,$\frac{\partial p_{2}^{*}}{\partial c}>0$，$\frac{\partial s^{*}}{\partial c}<0$.

## Proof of Theorem 4:

Compute the first-order partial derivatives of$s^{*}$with respect to$v_{1}$and$v_{2}$, respectively:

$$\frac{\partial s^{*}}{\partial v_{1}}=\frac{2p_{e}(a+h_{5}h_{3})[k^{3}B_{3}+k^{2}p_{e}^{2}B_{2}+kp_{e}^{4}B_{1}+p_{e}^{6}B_{0}]}{[4k^{2}(4-\alpha^{2})+p_{e}^{4}v_{1}v_{2}^{2}(1-\alpha^{2})(v_{1}-2v_{2}\alpha+v_{1}\alpha^{2})+4kp_{e}^{2}h_{10}]^{2}}$$

Which $B_{3}=-8(\alpha-2)(\alpha+2)^{2}$,$B_{2}=4(\alpha+2)[2v_{1}(v_{1}+2v_{2})+(\alpha^{3}+4\alpha^{2}-5\alpha-6)v_{2}^{2}]$,$B_{1}=2(\alpha^{2}-1)v_{2}^{2}[(\alpha^{3}+2\alpha^{2}+\alpha+4)v_{1}^{2}+2(\alpha+2)(\alpha^{2}+1)v_{1}v_{2}-4(\alpha+1)v_{2}^{2}]$,$B_{0}=v_{1}^{2}v_{2}^{4}(\alpha-1)^{2}(\alpha+1)^{2}(\alpha^{2}+1)$

Let $g_{v_{1}}(k)=k^{2}p_{e}^{2}B_{2}+kp_{e}^{4}B_{1}$ be given, when $g_{v_{1}}(k)=0$, we can get $k_{v_{1}}=\frac{-p_{e}^{2}B_{1}}{B_{2}}$. When $k>k_{v_{1}}$,$g_{v_{1}}(k)>0$,$\frac{\partial s^{*}}{\partial v_{1}}>0$.

$$\frac{\partial s^{*}}{\partial v_{2}}=\frac{4p_{e}(a+h_{5}h_{3})[k^{3}C_{3}+k^{2}p_{e}^{2}C_{2}+kp_{e}^{4}C_{1}+p_{e}^{6}C_{0}]}{[4k^{2}(4-\alpha^{2})+p_{e}^{4}v_{1}v_{2}^{2}(1-\alpha^{2})(v_{1}-2v_{2}\alpha+v_{1}\alpha^{2})+4kp_{e}^{2}h_{10}]^{2}}$$

Which $C_{3}=-4(\alpha-2)(\alpha+2)^{2}$,$C_{2}=(\alpha+2)[(\alpha^{3}-3\alpha-1)v_{1}^{2}+(-\alpha^{3}+\alpha+2)v_{1}v_{2}+(2-\alpha^{2})v_{2}^{2}]$,$C_{1}=v_{1}v_{2}(\alpha^{2}-1)[2\alpha(\alpha+1)^{2}v_{1}^{2}-(\alpha^{3}+4\alpha^{2}+5\alpha-2)v_{1}v_{2}-4\alpha(\alpha+2)v_{2}^{2}]$,$C_{0}=\alpha v_{1}^{2}v_{2}^{4}(\alpha^{2}-1)^{2}$

Let $g_{v_{2}}(k)=k^{2}p_{e}^{2}C_{2}+kp_{e}^{4}C_{1}$ be given, when $g_{v_{2}}(k)=0$, we can get $k_{v_{2}}=\frac{-p_{e}^{2}C_{1}}{B_{2}}$. When $k>k_{v_{2}}$,$g_{v_{2}}(k)>0$,$\frac{\partial s^{*}}{\partial v_{2}}>0$.
